# Causes of post-installation penetration of jack-up spudcan foundations in clays

**DOI:** 10.1371/journal.pone.0206626

**Published:** 2018-11-05

**Authors:** Yu Ping Li, Yu Yang, Jiang Tao Yi, Jia Hui Ho, Jian Yong Shi, Siang Huat Goh, Fook Hou Lee

**Affiliations:** 1 Key Laboratory of Ministry of Education for Geomechanics and Embankment Engineering, Geotechnical Engineering Research Institute, Hohai University, Nanjing, China; 2 Department of Civil and Environmental Engineering, National University of Singapore, Singapore, Singapore; 3 Key Laboratory of New Technology for Construction of Cities in Mountain Area(Chongqing University), Ministry of Education, School of Civil Engineering, Chongqing University, Chongqing, China; 4 Engineering Cluster, Singapore Institute of Technology, Singapore, Singapore; Universita degli Studi di Napoli Federico II, ITALY

## Abstract

This paper examines possible causes of additional spudcan settlement after preloading using both centrifuge model tests and small strain finite element analysis, in which spudcan settlement due to cavity collapse, consolidation settlement and settlement due to cyclic loading are incorporated. Back-analyses of seven jack-up rigs in the Gulf of Mexico show that even complete cavity collapse could only explain part of the measured additional settlements in the majority of the cases. Small strain finite element analyses also show that spudcan consolidation settlement is likely to account for even less of the additional settlement than cavity collapse in the sites considered. On the other hand, centrifuge model tests show that large amplitude cyclic rocking has a very significant effect on spudcan settlement, even if half of the preload has been removed. However, this effect cannot be explained by the exceedance of the yield envelope since the loading combination had not exceeded the yield envelope. One possible explanation is the stiffness and strength degradation of the soil under cyclic loading. In view of this, a conservative approach is recommended in instances where large amplitude cyclic rocking, such as that arising from storm loading, is expected shortly after preloading. The presence of lattice legs is found to reduce the spudcan settlement during large amplitude cyclic rocking.

## 1. Introduction

Bearing capacity of spudcan foundations for offshore mobile jack-up rigs is currently assessed using methods recommended by ISO [1]. These include methods recommended by Skempton [2], Houlsby & Martin [3] and Hossain & Randolph [4, 5]. However, large, rapid post-installation spudcan settlements have occasionally been reported before and after the removal of preloading. In many cases, the cause is spudcan punch-through failure occurred in stiff soil deposit overlying soft soil deposit [6, 7]. Recently, Menzies and Roper [8] reported additional spudcan settlements under the maximum preloading at seven locations in the Gulf of Mexico, which however did not appear to be caused by punch-through. They attributed this to an unaccounted-for loading possibly arising from soil “backflow” into the cavity. This postulate is, however, not proven. ISO [1] indicates that spudcan settlement may consist of four components, i.e. elastic settlement, consolidation settlement, settlement due to cyclic loading, and settlement due to seabed instability. The elastic settlement is the component which occurs immediately on loading application. This component is often not critical post-installation since it would have taken place upon application of preload. The other three settlement components are still not fully understood. Of these three, seabed instability-induced settlement is likely to be highly dependent upon specific site conditions.

This paper examines three components of the spudcan settlement, i.e elastic settlement due to cavity collapse, consolidation settlement and settlement due to cyclic loading, using both centrifuge model tests and finite element analyses. Most importantly, effects of lattice legs on these spudcan settlement components are incorporated.

## 2. Field data study-settlement due to cavity collapse

The effect of cavity collapse on spudcan settlement is first estimated for seven sites reported by Menzies and Roper [8]. As shown in Table 1, the reported additional spudcan settlements range from 0.13 to 0.31 times the spudcan diameter D, while maintaining the maximum preload in seven Gulf of Mexico sites. In this study, cavity depths after the completion of spudcan preloading were estimated using Hossain et al.'s [9] method and Li et al.'s [10] method. The former method does not consider the effect of lattice leg whilst the latter does. In Li et al.'s [10] method, the cavity depth within the confines of the lattice leg is dependent on the area and opening ratios of the lattice leg. The area ratio *A*_*a*_ is defined as the ratio of the cross-sectional area of lattice leg to the maximum spudcan footprint, while the opening ratio *e* is defined as the ratio of the area of the openings at the sides of the lattice leg to the total area of the lattice sides. The lattice leg configurations in the seven sites were not specified by Menzies and Roper [8]. However, Li et al. [10] noted that area and opening ratios typically range from 0·3 to 0·7 and 0·6 to 0·8, respectively, for three- and four-chorded lattice legs. Hence, a lower- and upper-bound area ratio of 0.3 and 0.7 were assumed herein. The opening ratio was assumed to be 0.75 since the range of its variation is much smaller.

**Table 1:** Comparison between back-calculated settlements due to full cavity collapse and the measured additional settlements in seven Gulf of Mexico sites.

Site	Penetration depth d (m)	Spudcan diameter D (m)	Soil strength s_u_ (kPa)	Predicted cavity depth (m)					Back-calculated normalized settlements due to full cavity collapse *w*_*cav*_ / D		Measured maximum additional settlements w/D
				Hossain *et al*. (2005) *H*_*max*_	Hossain H_ave_ = 0.43H_max_^1^	Li *et al*. (2017(a)) *H*_*i*_^2^	Li *et al*. (2017(a)) *H*_*ave*_ = H_i_ A_a_	H_o_ = 0.22D^3^	Hossain *et al*. (2005)	Li *et al*. (2017(a))	
1	37.5	13.5	2.4+1.35z^4^	3.4	1.5	3.9–4.0	1.2–2.8	3.0	0.04	0.06–0.10	0.15
4	14.2	12.0	19.2+1.46z	5.8	2.5	6.6–6.6	2.0–4.7	2.6	0.08	0.10–0.18	0.23
5	17.3	12.0	15.6+1.24z	5.5	2.4	6.6–6.3	1.9–4.4	2.6	0.09	0.10–0.18	0.23
7	22.3	12.0	5.7+1.18z	3.7	1.6	4.3–4.3	1.3–3.0	2.6	0.06	0.08–0.13	0.23
8	23.6	12.0	8.6+1.02z	4.2	1.8	4.8–4.9	1.4–3.4	2.6	0.08	0.09–0.17	0.31
9	8.4	12.0	23.0+1.26z	6.1	2.6	6.9–7.0	2.1–4.9	2.6	0.12	0.13–0.24	0.13
12	10.3	14.6	18.2+2.09z	7.3	3.1	8.3–8.4	2.5–5.8	3.2	0.29	0.31–0.41	0.17

^1^ Volume-averaged cavity depth H_ave_ is determined assuming a vertical inclined angle of 36^0^ for the cavity slope according to Hossain et al. (2005)’s PIV test results.

^2^ The predicted lower and upper cavity depths are due to different area ratios of lattice leg being assumed, lower and upper area ratios A_a_ of 0.3 and 0.7 are used here, respectively. For both cases, a typical lattice leg opening ratio e of 0.75 is assumed.

^3^ Based on Li et al. (2017(a)), an average cavity outer depth (i.e. the cavity outside of the lattice leg) H_o_ of 0.22D is used for spudcan enclosed with typical lattice leg (A_a_ = 0.6, e = 0.75) to determine the outer cavity volume within the spudcan footprint.

Hossain et al. [9] proposed a cylindrical cavity shape to be used. However, their centrifuge observations showed that cavities for spudcans without lattices in normally consolidated soil have an inverted shallow conical shape. This was also noted by Li et al. [10]. For this reason, a shallow conical cavity is assumed instead to evaluate the cavity volume when Hossain et al.'s [9] method is used. For spudcan with lattices, Li et al. [10] noted that, within the confines of the lattice leg, the cavity is almost vertical and co-planar with the sides of the lattice cage. Outside the confines of the lattice legs, the cavity is much shallower and the gradient of the cavity wall is also gentler than that inside the lattice leg. Since the lattice leg is unlikely to have significant effect on the cavity outside it, the shape of the cavity outside the lattice is assumed to be the same as that without the lattice, which is a shallow inverted cone [9, 10].

**Fig 1:**
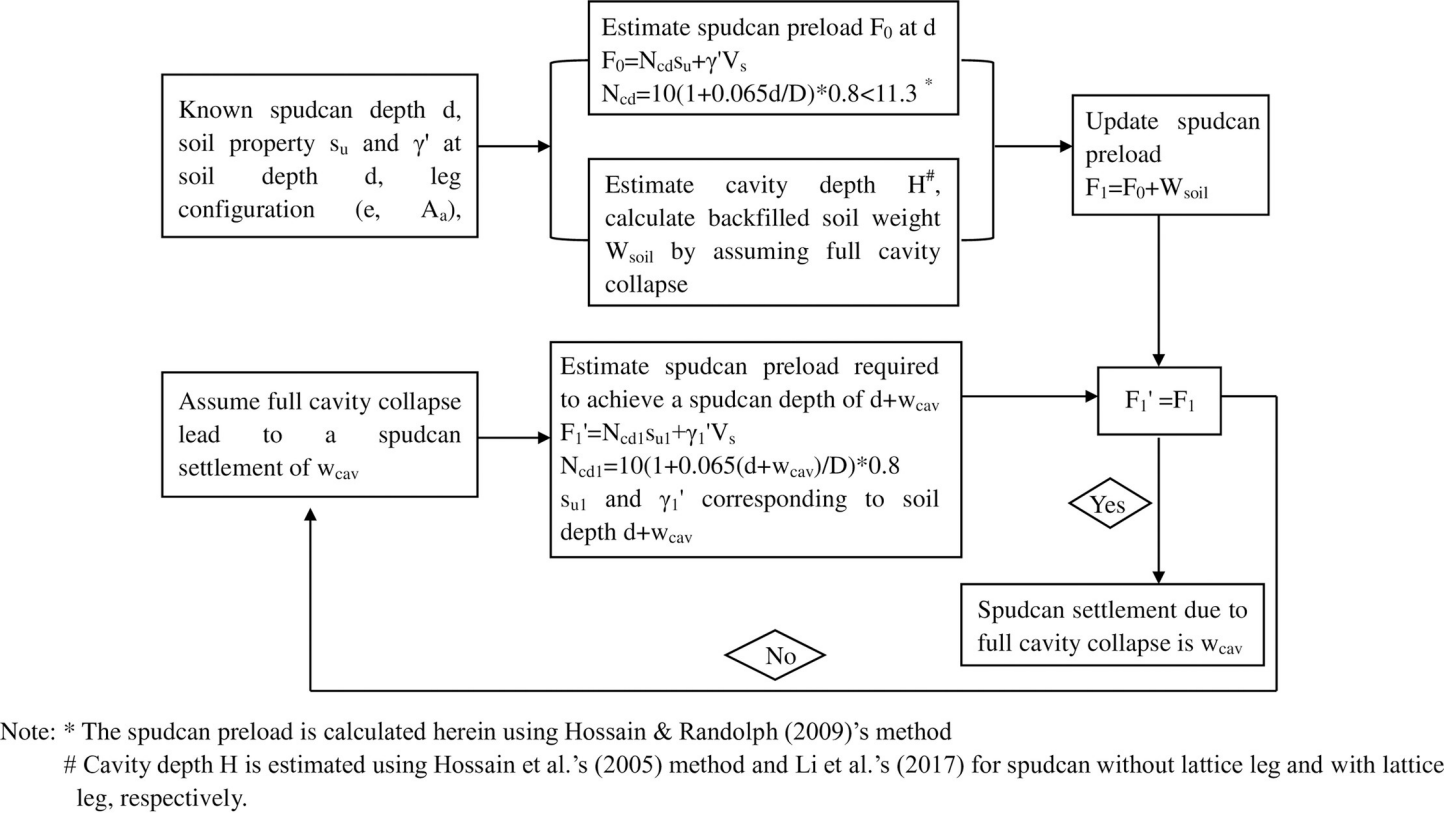
Flow chart of spudcan settlement back-calculation due to full cavity collapse.

By assuming that the cavity volume calculated above is completely back filled, the induced additional loads can be used to back-calculate the additional settlement using an appropriate spudcan bearing capacity method. Menzies and Roper [8] noted that Hossain & Randolph's [4] method provides an upper bound of the observed bearing capacity, whereas Skempton's [2] and Hansen's [11] methods give good average prediction of the thirteen sites examined by Menzies and Roper [8]. If Hossain & Randolph's [4] bearing capacity factor was 20% reduced, then it gives very similar prediction to Skempton's [2] and Hansen's [11] predictions. Following Hossain and Randolph [5], the spudcan bearing capacity was estimated using Hossain & Randolph's [4] bearing capacity factor with a 20% reduction to back-calculate the additional settlement due to cavity collapse. The detailed back-calculating process on spudcan settlement due to full cavity collapse has been described in a flow chart in Fig 1.

**Fig 2:**
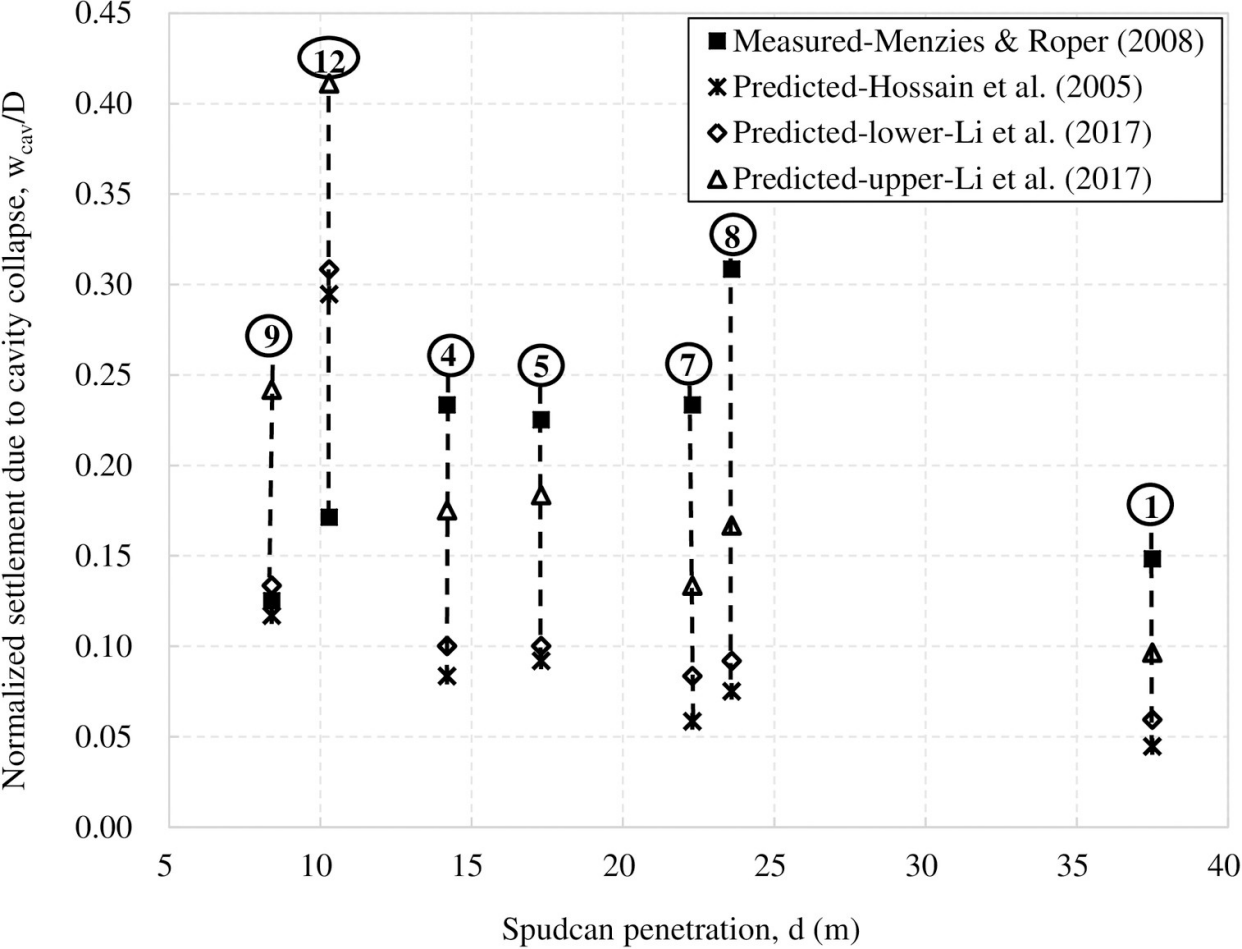
Comparison between back-calculated settlements due to full cavity collapse and the measured additional settlements in seven Gulf of Mexico sites.

As shown in Table 1 and Fig 2, the back-calculated settlements due to backfill of cavity volume are generally smaller than measured settlements except for Sites 9 and 12. For Site 9, the back-calculated settlement using Hossain et al.'s [9] cavity depth lies just below the measured settlement; while that obtained using Li et al.'s [10] lower-bound cavity depth is also in good agreement with the measured data. For Site 12, using Hossain et al.'s [9] and Li et al.'s [10] cavity depth formulae lead to settlement values which significantly exceed the measured data. As Table 1 shows, with the exception of Sites 9 and 12, spudcan penetration depths in other five sites are much larger than the stable cavity depths estimated by Li et al. [10]. On the other hand, for Sites 9 and 12, spudcan penetration depths are only slightly larger than stable cavity depths. Given the uncertainty in the area and opening ratios and its effect on the stable cavity depth, it is possible that, in these two sites, the stable cavity depth might not have been reached post-installation. For instance, for Site 12, Li et al.'s [10] method estimates a stable cavity depth of about 8.3m, which implies that the spudcan depth required for stable cavity formation must be larger than this depth. In this Site, the spudcan penetration depth is 10.3m, this implies that the distance between the mudline and the top of spudcan is only about 6.1m. Hence, the final stable cavity might have not been formed at this Site at full preload. Using the embedment depth of spudcan top as the cavity depth gives a better agreement with the measured additional settlement in Site 9, which however still exceeds the measured settlement in Site 12. This may suggest that the cavity is not fully backfilled in Site 12.

The above discussion would suggest that cavity collapse may be able to account for the measured additional settlements in Sites 9 and 12. On the other hand, cavity collapse is able to account for only part of the measured additional settlements in the other five sites. Compared with the back-calculated settlements using Li et al.'s [10] method, the remaining measured settlements of approximately 0.05D to 0.22D in the five sites are likely to be due to other causes.

## 3. Consolidation settlement

### 3.1 Numerical methodology

**Fig 3:**
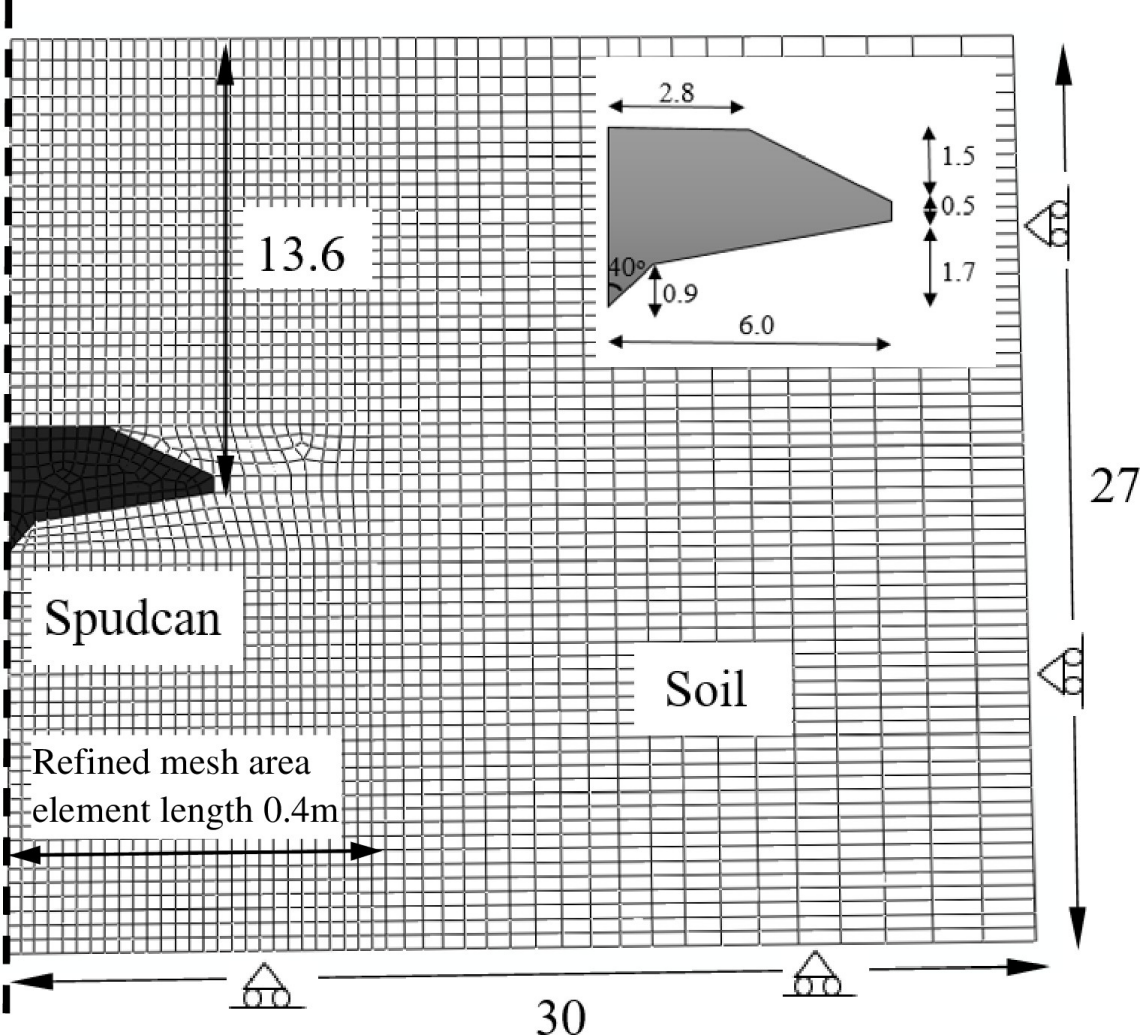
The 2D axisymmetric finite element mesh (unit: M).

Coupled consolidation small strain finite element analysis was conducted in ABAQUS/Standard version 6.14 to assess the consolidation settlement of the spudcan in a single clay layer, in which effect of spudcan consolidation load ratio was examined. Spudcan consolidation load ratio is defined as the ratio of the vertical load hold during consolidation over the maximum preload. Fig 3 shows the two dimensional (2D) axisymmetric finite element mesh. To validate against the centrifuge model test [12], the soil geometric dimensions (27m in depth and 30m in radial extent) and boundary conditions adopted here were consistent with those used in the test. As shown in Fig 3, soil displacement normal to the axisymmetric line and the right vertical line was constrained, soil flow in the vertical direction was also constrained to be zero at the soil base. Drained was only allowed at the top of the soil surface. A finer mesh (element length 0.4m) was used one spudcan diameter laterally from spudcan center while a coarse mesh was used far away. This mesh density was found to yield consistent spudcan consolidation settlements. Four-node axisymmetric quadrilateral bilinear displacement and pore pressure elements were used for the soil domain. The soil was modelled using a modified Cam-clay (MCC) material, MCC parameters shown in Table 2 are consistent with soil properties used in the centrifuge model test [12]. By using Wroth’s [13] Eq (1), the simulated soil undrained shear strength *s*_*u*_ = 1.46*z* kPa could be obtained from MCC parameters. The soil was fully saturated with its initial stress being prescribed under a *K*_*0*_ condition, in which *K*_0_ = 1 − sin *φ*' is an earth pressure coefficient at rest and *φ*' is a soil critical state friction angle. The initial average void ratio and average density were set to be 1.3 and 1.06, respectively.

(suσv')NC=(sinφ'2a)(a2+12)λ−κλ (1)

Where $a=\frac{3-\sin\varphi '}{2(3-2\;\sin\varphi ')}$, *σ*_*v*_' is soil effective vertical stress, *λ* and *κ* are the slopes of soil isotropic compression and swelling lines.

**Table 2:** Parameters used in the modified Cam-clay model.

Slope of critical state line *M*_*2*_	0.9
Slope of isotropic normal compression line *λ*	0.244
Slope of isotropic swelling and re-compression line *κ*	0.0523
Specific volume of soil at critical state (*p*' = 1*kPa*) Γ	3.221
Effective Poisson’s ratio *υ*'	0.33
Effective unit weight *γ*'(*kN*/*m*^3^)	6.0
Coefficient of earth pressure at rest *K*_*o*_	0.6
Coefficient of permeability *k* (m/s)	2.0×10^-8^
Soil critical state friction angle *φ*'	23^0^

Noted that the modelled soil strength falls to be the lower bound of the soil strength profiles in the Gulf of Mexico sites [8]. Soil bed with a lower soil undrained shear strength is usually correlated with a higher water content [14], and hence a higher void ratio. In view of this, spudcan embedded at soil with a lower undrained shear strength is likely to have a larger consolidation settlement. The soil strength profile modelled here would hence provide an upper bound estimation on spudcan consolidation settlements in the Gulf of Mexico sites.

**Fig 4:**
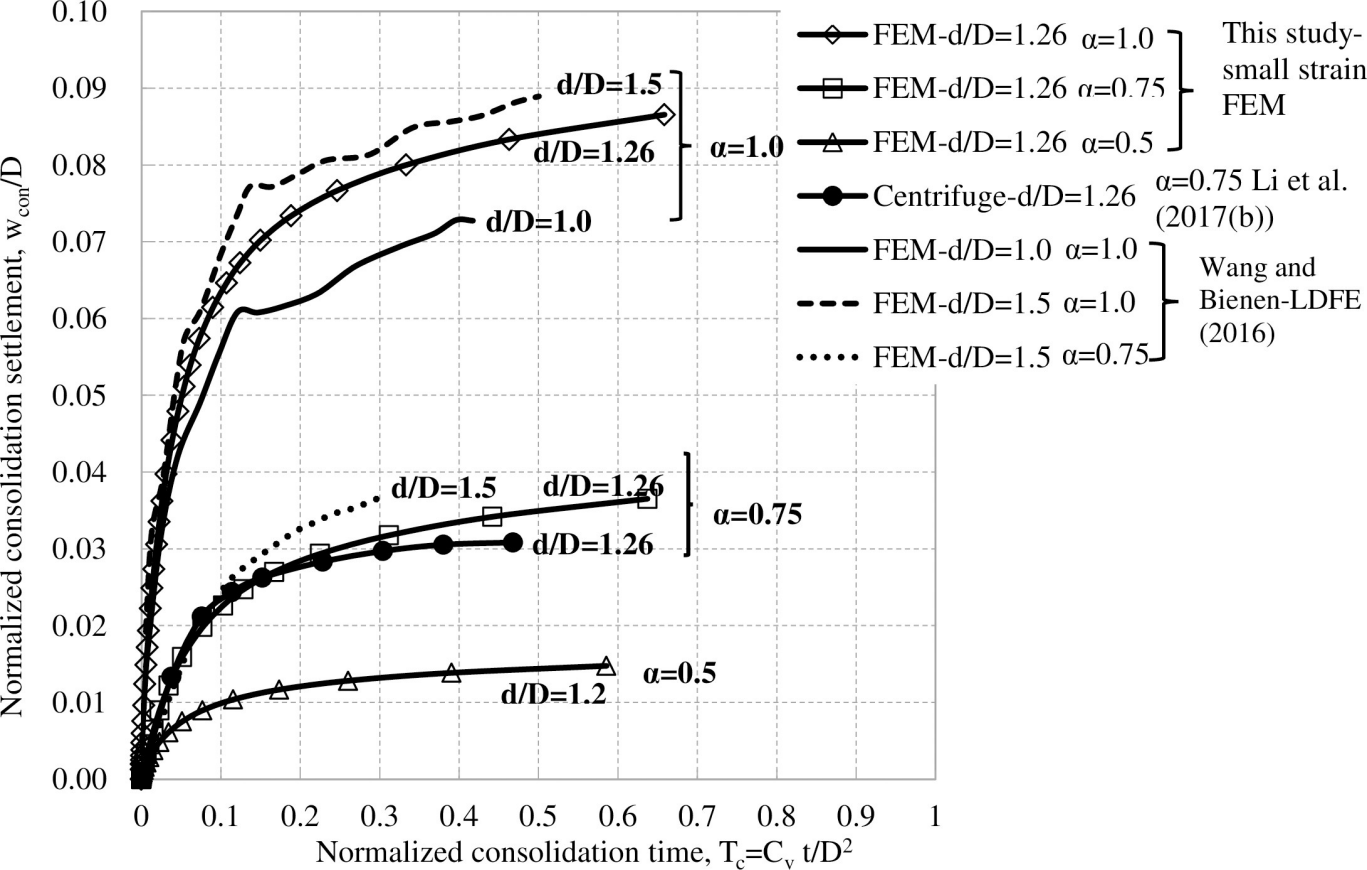
Comparison of measured and computed consolidation settlement for spudcan without leg in clay.

To examine spudcan post-installation settlements during jack-up rig operation period, three stages were involved in the numerical simulation, i.e. penetration-unloading-consolidation. The first two stages were usually completed under undrained condition. In view of this, total time for each of the two stages was set to be 1000s, which is relatively short and not likely to induce significant soil consolidation. A total time of 1.6e8s (5 years) was set for the consolidation stage to allow full consolidation. In this numerical simulation, a full-Newton method was used as the convergence criteria. Following Templeton’s [15] method, the spudcan was firstly wished into a position (Fig 3, *d* = 13.6 m) at a small distance above the target depth. It was then penetrated over a distance of 1.5 m to achieve plastic penetration and establish the penetration stress field beneath the spudcan. The spudcan was then unloaded to a prescribed consolidation load ratio to commence the consolidation stage. Li *et al*. [12] noted that this method gives a good estimation of the measured post-consolidation penetration resistance and consolidation settlement when compared with centrifuge model test data, the latter will be further illustrated in Fig 4.

### 3.2 Numerical result analysis

The working load for jack-up rig is typically half of the maximum preload; this corresponds to a consolidation load ratio of 0.5. On the other hand, Menzies and Roper’s [8] observations were made at a consolidation load ratio of 1.0. To cover this range, three consolidation load ratios 1.0, 0.75 and 0.5 were examined herein. Fig 4 compares the computed consolidation settlements versus normalized consolidation times *T*_*c*_ *= c*_*v*_*t/D*^*2*^ at different consolidation load ratios with other results. *c*_*v*_ is the soil vertical coefficient of consolidation surrounding spudcan consolidation depth, which is about 40m^2^/year. Fig 4 shows that the computed consolidation settlement agrees well with Li et al.'s [12] centrifuge test result at a spudcan consolidation depth of d/D = 1.26 and a consolidation load ratio α of 0.75. Furthermore, the computed consolidation settlements compare well with Wang and Bienen's [16] large deformation finite element (LDFE) results at consolidation load ratios of 0.75 and 1.0, respectively. This would suggest the feasibility of this numerical method on estimating spudcan consolidation settlement. Wang and Bienen’s [16] LDFE results also show that spudcan consolidation settlement is also influenced by spudcan embedment depth, which is however much less significant as that of consolidation load ratios. Bienen and Cassidy’s [7] centrifuge test results also didn’t show spudcan embedment depths effect on consolidation settlements. In this respect, spudcan embedment depth effect will not be studied here.

Fig 4 illustrates that a larger consolidation load ratio tends to yield a larger consolidation settlement. For each consolidation load ratio, the consolidation settlement increases rapidly at early consolidation stage and finally converges to a constant value. Take spudcan settlement under the consolidation load ratio of 1.0 for instance, about 50% of the consolidation settlement, that is about 0.05D, has taken place by *T*_*c*_ = 0.04 (for *c*_*v*_ = 40m^2^/year and *D* = 12m, the corresponding consolidation time is *t* = 54 days). This consolidation settlement magnitude at *T*_*c*_ = 0.04 is equal to the minimum of the excess settlement (ranging from 0.05D to 0.22D) which could not be accounted for by cavity collapse. For offshore sites with a lower coefficient of consolidation, a much longer consolidation time *t* will be needed to reach this settlement magnitude. In the Gulf of Mexico sites, additional spudcan settlements were however monitored upon the completion of preloading. This, together with that the computed settlement falls to be an upper bound site estimation, consolidation settlement is likely to explain only a small part of the measured additional settlements by Menzies and Roper [8].

## 4. Settlement due to cyclic rocking

### 4.1 Centrifuge model test description

Yang *et al*. [17] and Yang [18] reported centrifuge model test data relating to spudcan settlement due to cyclic rocking in normally consolidated (NC) Malaysia kaolin clay (Table 2). Their centrifuge tests were conducted under a model gravity of 100g. The spudcan was penetrated into the consolidated soil bed with a constant velocity of 0.6mm/s to an equivalent prototype soil depth of about 15m (1.25D). Based on Finnie & Randolph’s [19] dimensionless velocity, this rate of penetration is sufficient to ensure undrained condition during spudcan penetration. The spudcan load was then reduced to 50% of its preload at that depth, to simulate removal of preload. For “no-dissipation” tests, spudcan cyclic rocking commenced immediately after preload removal; this simulates the condition wherein the spudcan is subjected to environmental loads in the offshore field. For “full-dissipation” tests, soil surrounding spudcan footing was allowed to consolidate under the remaining vertical load to allow a full dissipation of excess pore pressure. Two rocking amplitudes were used. The first is a small amplitude rocking with angular amplitude of 0.2°, which represents low-level environmental loading that is unlikely to cause large-scale yielding of the spudcan foundation. The second is a large-amplitude rocking with angular amplitude of 2.3° (spudcan lateral distance around 1m) which represents larger wind and wave loading.

**Fig 5:**
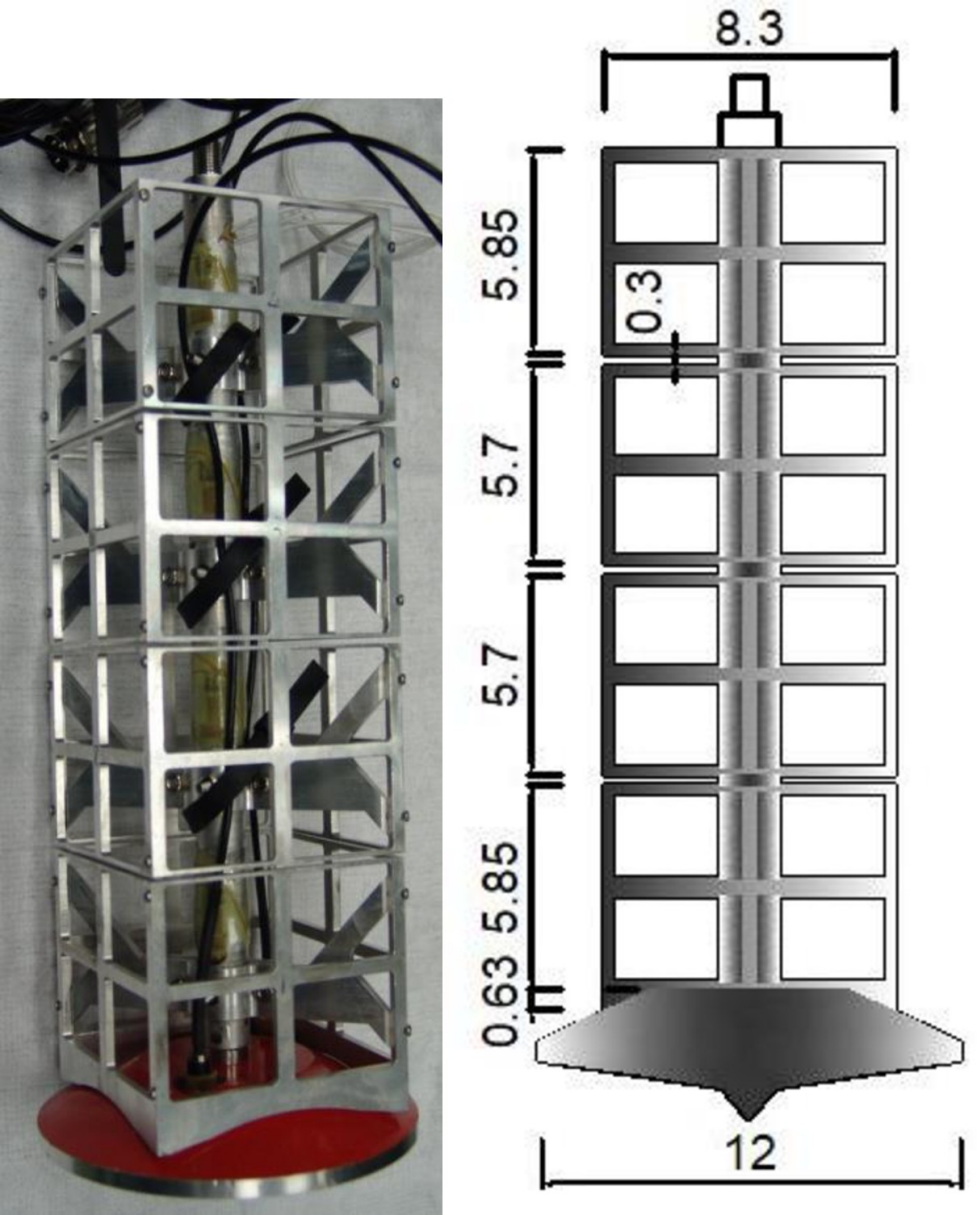
Model lattice leg configuration and its elevated dimensions in prototype scale (unit: M).

Table 3 includes a selection of tests reported by Yang et al. [17]. As this Table shows, Yang et al.'s [17] lattice legs have opening ratio of 0.75, which is also within the typical range of values of opening ratios used in prototype legs [10]. Fig 5 illustrates the typical model lattice leg configuration and its dimensions, which has an opening ratio of *e* = 0.75 and an area ratio of *A*_*a*_ = 0.6. Yang et al. [17] also tested model spudcans with fully enclosed sleeves, but these will not be analyzed herein since they do not bear close resemblance to prototype legs.

**Table 3:** Centrifuge testing program in normally consolidated Malaysia kaoline clay.

Testing No	Opening ratio	Area ratio	Leg shape	Dissipation before cyclic rocking	Amplitude (°)	Number of cycles	Rocking settlement	V_ult_ at 1.3D (MN)	M_1_ at 1^st^ cycle (MNm)^1^	M_1_/DV_ult_
SN1	1	0	/	No dissipation	0.2	1000	0.030D	29	17.02	0.05
SN2	1	0	/	Full dissipation	0.2	1000	0.005D	29	22.91	0.07
RO1	0.75	0.61	Square	No dissipation	0.2	1000	0.029D	31	/	/
RO2	0.75	0.61	Square	Full dissipation	0.2	1000	0.002D	31	/	/
SN3	1	0	/	Full dissipation	2.3	70	0.076D	29	45.3	0.13
SN4^2^	1	0	/	No dissipation	2.3	2	/	29	16.35	0.05
RO3	0.75	0.61	Square	Full dissipation	2.3	70	0.077D	31	55	0.15
CO3	0.75	1.0	Circular	Full dissipation	2.3	70	0.071D	31	33.5	0.09
CO4	0.75	1.0	Circular	No dissipation	2.3	30	0.127D	31	14.8	0.04

^1^The bending moment measured from the straingage installed at the lowest place of the shaft, closing to the spudcan footing, which is roughly assumed to be the moment experienced by the spudcan footing.

^2^Test SN4 was terminated after 2 cycles of loading owing to excessive settlement.

Prefixes

• SN refers to spudcan model without lattice leg

• RO refers to spudcan model with square cross section lattice leg with opening ratio of 0.75 and area ratio of 0.61

• CO refers to spudcan model with circular cross section lattice leg with with opening ratio of 0.75 and area ratio of 0.61.

Suffix numerals

• 1 refers to “no-dissipation” test with small amplitude rocking

• 2 refers to “full-dissipation” test with small amplitude rocking

• 3 refers to “full-dissipation” test with large amplitude rocking

• 4 refers to “no-dissipation” test with large amplitude rocking.

### 4.2 Spudcan settlement under small amplitude rocking

**Fig 6:**
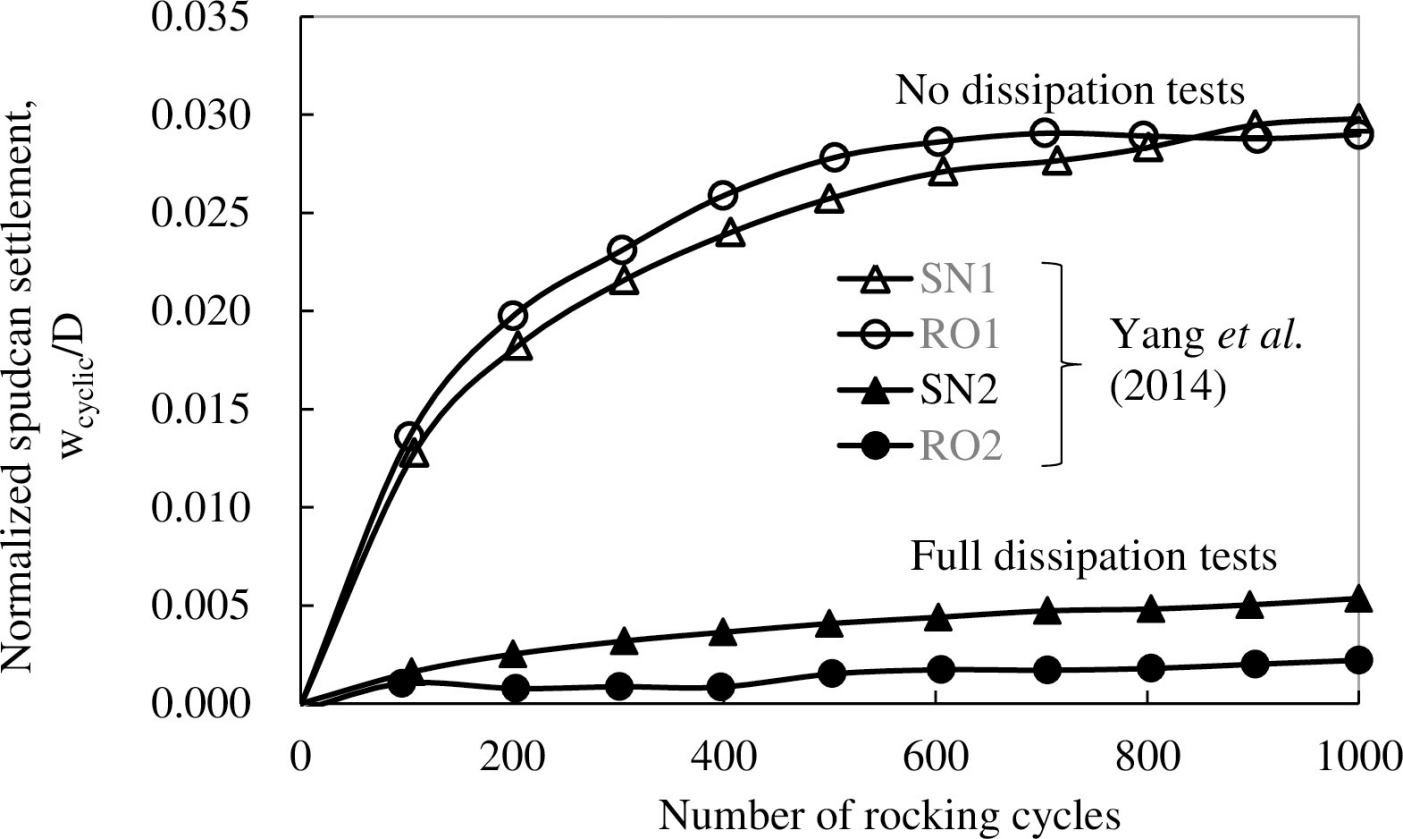
Normalized spudcan settlement during small amplitude cyclic rocking in NC clay.

Fig 6 plots normalized spudcan settlements during small amplitude rocking [17]. As can be seen, for both spudcans with and without lattice legs, settlements increase gradually with the numbers of cycles and approach stable values after about 1000 cycles. The “no-dissipation” tests show much larger settlements than those of “full-dissipation” tests, and the settlement differences between “no-dissipation” and the corresponding “full-dissipation” tests are about 0.026*D* and 0.025*D* for spudcans with and without lattice legs, respectively. These values are somewhat larger than, but of the same order as the computed consolidation settlement of 0.015*D* at a consolidation load ratio of *α* = 0.5 and a similar consolidation depth of 1.26*D*, Fig 4. Furthermore, the lattice leg does not significantly influence spudcan settlement under small amplitude rocking.

**Fig 7:**
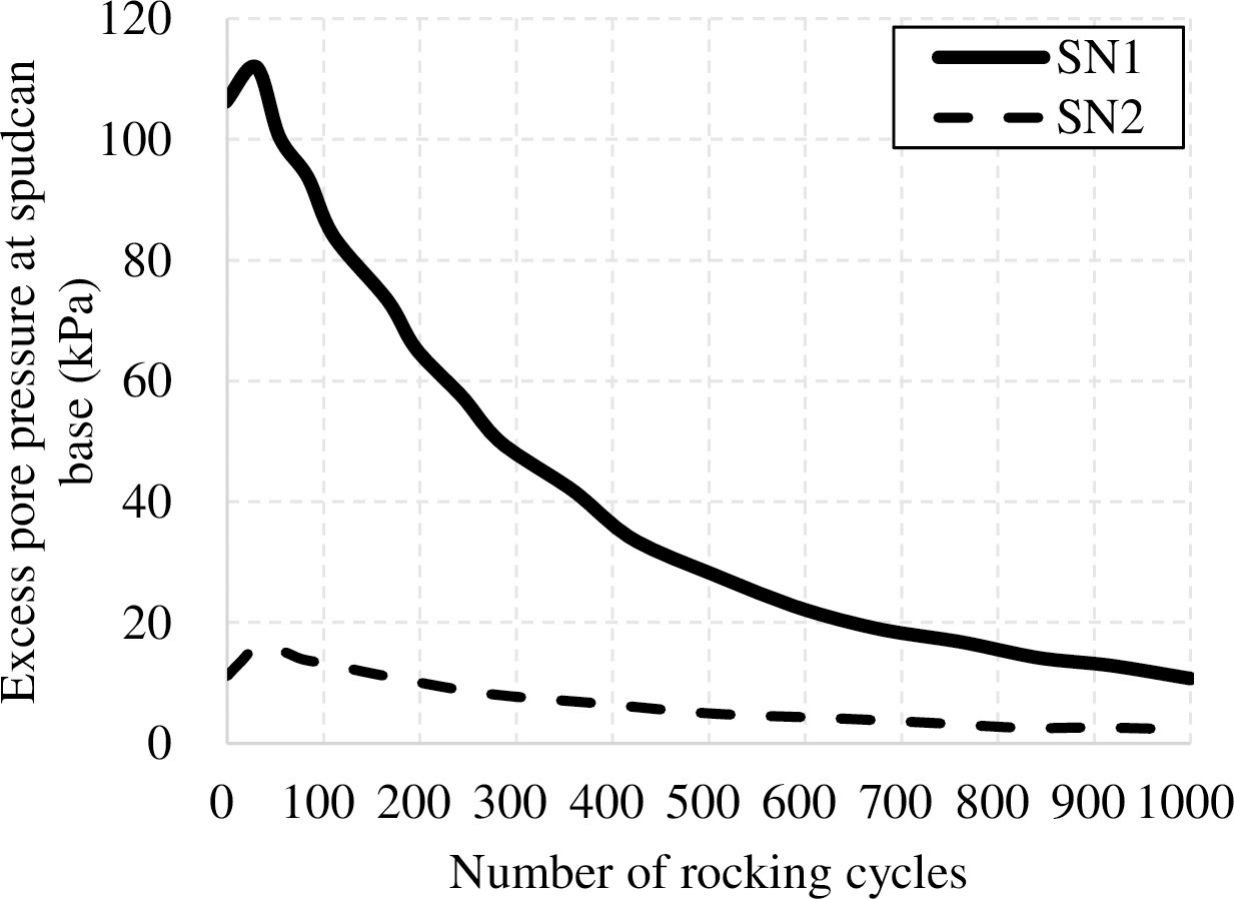
Excess pore pressure at spudcan base during small amplitude cyclic rocking in NC clay.

Fig 7 illustrates Yang et al.'s [17] pore pressure dissipation data, which shows a small increase in excess pore pressure over the first ~20cycles of rocking, followed by monotonic decrease. This indicates that while there might have been some initial increase in excess pore pressure due to rocking, the amount is relatively small and pore pressure evolution is dominated by the decrease in excess pore pressure from an initially high value caused by spudcan penetration. This would suggest that much of the measured settlement differences between “no-dissipation” and “full-dissipation” tests can be explained by consolidation during the rocking episode. There might have been a small amount of cyclic-induced settlement, but this is generally small, typically between 0.03D and 0.005D, and are much smaller than the observed spudcan settlement increments reported by Menzies and Roper [8].

### 4.3 Spudcan settlement under large amplitude rocking

**Fig 8:**
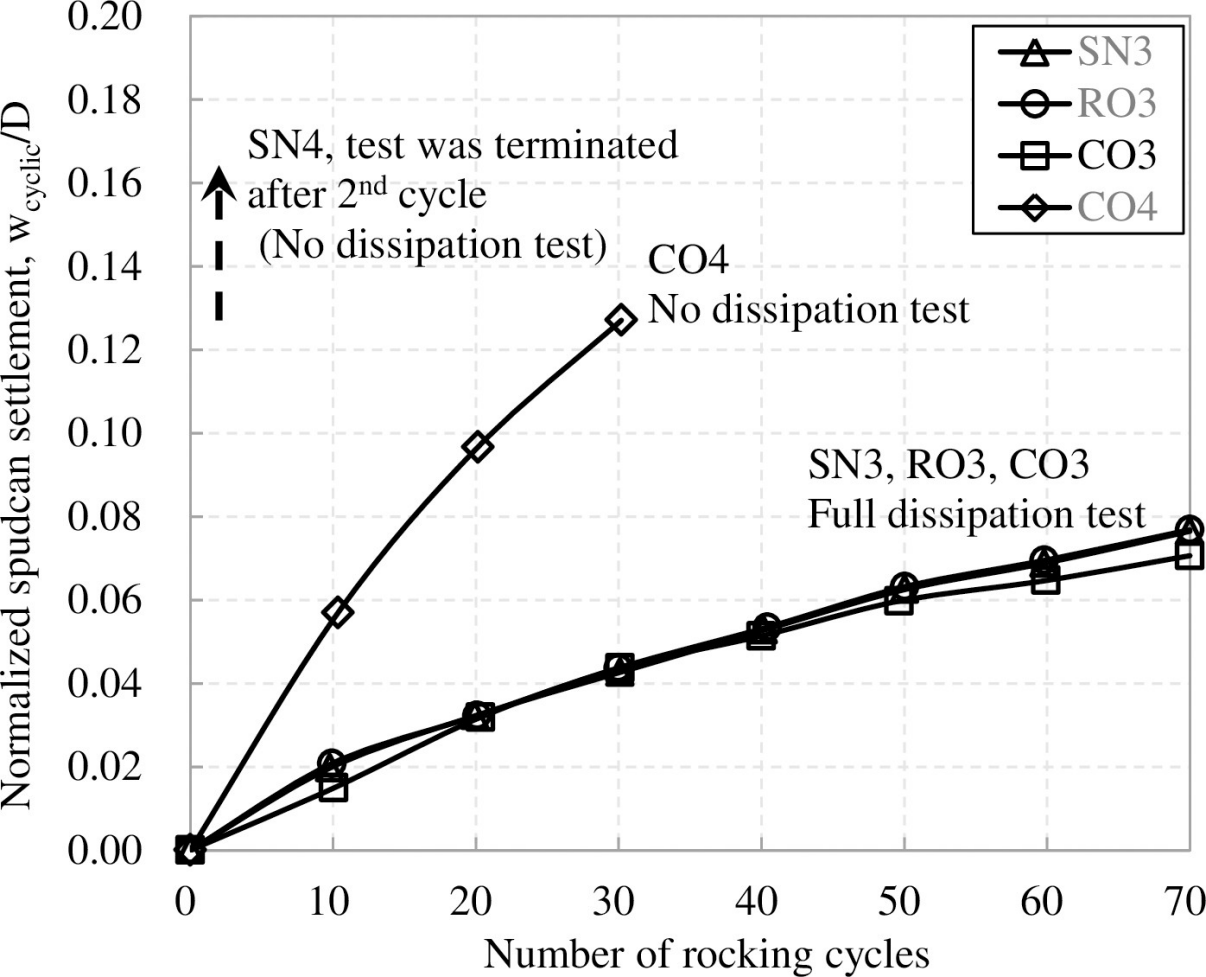
Normalized spudcan settlement during large amplitude cyclic rocking in NC clay (after Yang et al. (2014)).

Spudcan settlement under large amplitude rocking is manifestly different from that under small amplitude rocking. As shown in Fig 8, spudcan settlement increases rapidly with successive cycles of rocking until the vertical movement reaches the limit of the centrifuge modelling equipment. Settlement had clearly not stabilized at this point. For “full-dissipation” tests, after 70 cycles, additional settlements ranging from 0.071D to 0.077D were recorded for spudcans with and without lattice legs. For “no-dissipation” tests, the additional settlement increases to 0.13D after 30 cycles for spudcan with lattice leg (CO4). In test SN4, settlement exceeds the equipment limit just after 2^nd^ cycles. This magnitude of settlement is much larger than that incurred under 1000 cycles of small amplitude rocking, and can explain the discrepancy range of 0.05D to 0.22D between back-calculated and measured settlements in cases 1, 4, 5, 7 and 8 reported by Menzies and Roper [8].

For “full-dissipation” tests, to distinguish the contribution of possible cavity collapse on spudcan settlements, back-calculation of spudcan settlements due to cavity collapse for spudcans with square lattice leg (RO3) and full circular lattice leg (CO3) were carried out. Using Li et al.'s [10] method, averaged cavity depths of 0.57m and 0.91m are obtained for the two cases in NC clays, respectively. Full cavity collapse would lead to additional spudcan settlements of about 0.02D and 0.03D for RO3 and CO3, respectively. Compared to their measured settlements of 0.077D and 0.071D (Table 3), there remains a significant proportion of settlements which cannot be adequately explained by cavity collapse. Furthermore, the durations of the large amplitude tests were much shorter than those of the small amplitude tests, owing to much smaller number of cycles. As Fig 7 show, the amount of pore pressure dissipation which occurs over the first 70 cycles are relatively insignificant and the spudcan foundation is still in a relatively undrained condition. Hence, consolidation is also unlikely to be a cause. Various other factors have been postulated, such as soil degradation effects [1, 20].

The settlement discrepancy between full dissipation test (CO4) and no dissipation test (CO3) is about 0.09D at 30 cycles for spudcan with full circular lattice leg. This discrepancy is much larger than the computed consolidation settlement of 0.015D at a load ratio α of 0.5. Furthermore, undrained condition should still prevail over the first 30 cycles, so much of this settlement discrepancy is likely to be caused by cyclic rocking rather than consolidation.

As Fig 8 shows, for full dissipation test, the lattice leg does not significantly influence spudcan settlement. However, comparison of SN4 and CO4 suggests that, for no dissipation test, the presence of the lattice leg appears to reduce spudcan settlement and enhance spudcan stability significantly.

**Fig 9:**
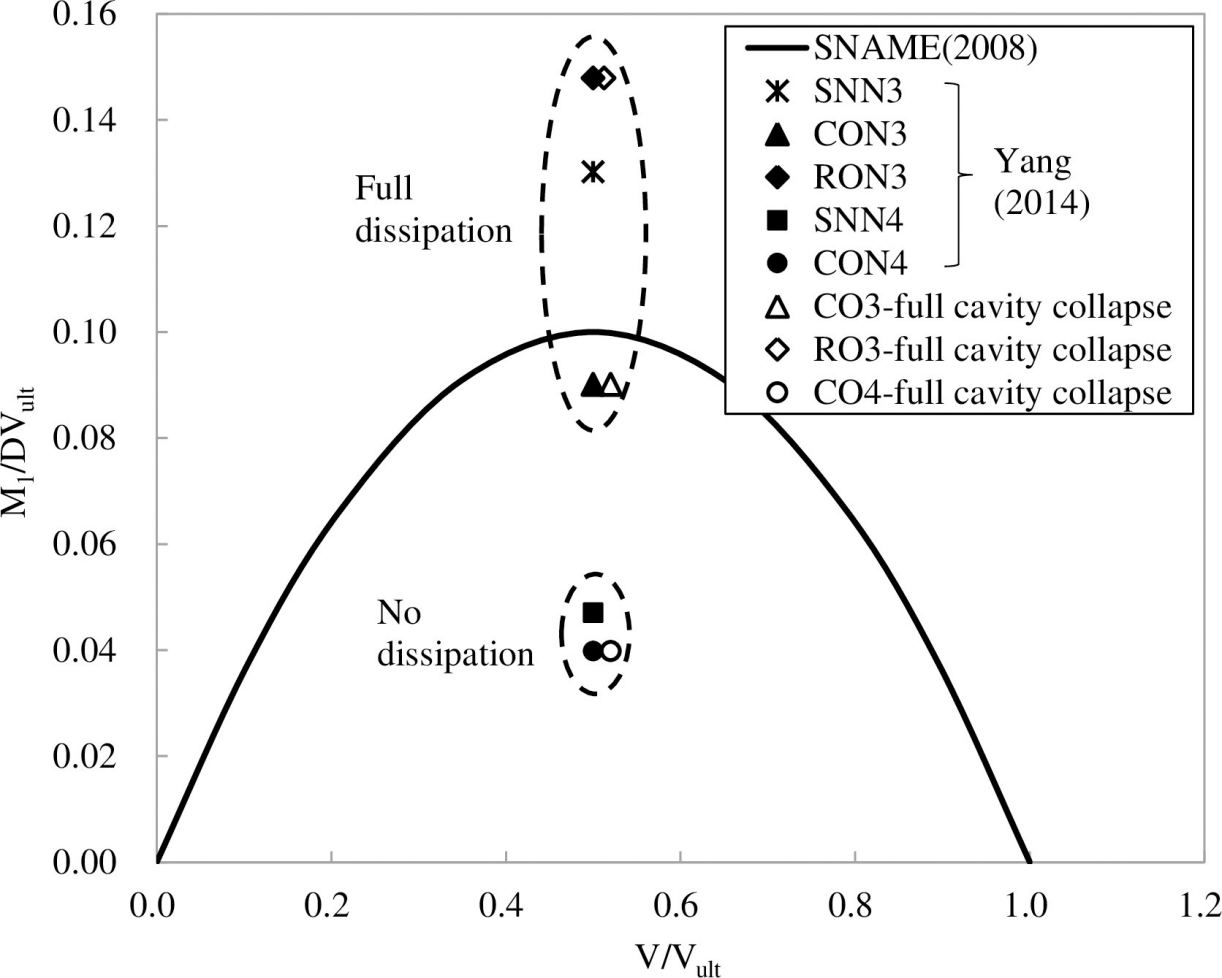
Comparison of spudcan bending moment after the 1^st^ cycle of rocking.

In Yang's [18] spudcan model, a full-bridge strain gauge was installed along the central shaft near the spudcan to measure the bending moment. Fig 9 plots the measured spudcan bending moment under the large amplitude cyclic rocking, together with the moment-vertical load yield envelope from SNAME [21], using the maximum preload as the ultimate vertical load *V*_*ult*_. For “no-dissipation” tests, the maximum bending moment is roughly half of the yield moment at the load ratio of 0.5. The spudcan bending moment appears to be slightly reduced by the presence of the full circular lattice leg (CO4), which is consistent with the response of the spudcan settlement as discussed above. Taking into account the increase in vertical loading in the event of cavity collapse leads to only a slight right-shift in the corresponding point. Hence, large settlements observed by Yang et al. [17] in “no-dissipation” tests cannot be readily attributed to exceedance of the yield locus. For “full-dissipation” tests, the maximum moment is larger, ranging from just below to about 1.5 times the yield moment. However, since the ultimate vertical load *V*_*ult*_ relates to that before consolidation, the increase in the post-consolidation ultimate vertical load [10, 22] may also lead to an increase in moment capacity and therefore size of the yield locus.

## 5. Implications of results

Menzies and Roper’s [8] field observations showed that large additional spudcan settlements can occur at maximum preloads. Part of these additional settlements can be explained in terms of cavity collapse. However, in Sites 1, 4, 5, 7 and 8, full cavity collapse and consolidation settlement appear to be unable to account for the additional settlement. Moreover, there is still an outstanding question on what caused the cavity collapse.

The issue of additional settlement was also examined by Yang et al. [17] using centrifuge models in which spudcans were subjected to rocking perturbation. Yang et al.'s [17] models were conducted under a load ratio of 0.5 and therefore cannot be compared directly with Menzies and Roper’s [8] cases. Nonetheless, Yang et al.'s [17] results still contain some significant qualitative indicators. Firstly, small amplitude rocking leads to relatively small settlement which is not only unlikely to explain the large settlement but is also much less than the settlement that would have resulted from cavity collapse. Hence, cavity collapse may not readily occur under small amplitude rocking. Secondly, Yang et al.'s [17] data shows that large-amplitude rocking can lead to large settlement over relatively small number of cycles. Although the measured settlement is still smaller than that observed by Menzies and Roper [8], this is due to limitation of the equipment, which was unable to track larger displacements. As Fig 9 shows, in spite of the large settlement, the maximum moment-vertical load combination for the no-dissipation cases lie well within the yield locus. Hence, by all accounts, the model should have been well-within operating envelope. Fig 9 shows that if the additional load from cavity collapse is added, the load point shifts rightwards slightly but still remains well within the yield locus.

**Fig 10:**
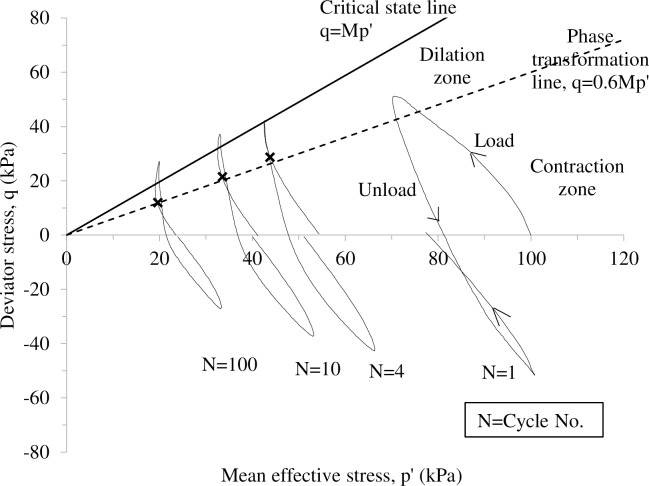
Typical phase transformation from contractive to dilative behaviour observed in normally consolidated Malaysia kaolin clay (after Ho (2014)).

The above discussion suggests that, even if the load point is within the yield locus, large amplitude cyclic rocking can still lead to large spudcan settlement. One possible reason may be the build-up cyclically generated excess pore pressure surrounding the spudcan footing, leading to a loss of soil effective stress, and thereby soil strength and stiffness. Ho [23] reported that cyclic triaxial loading can lead to a significant loss of effective stress if the cyclic stress ratio (q/p') exceeds a phase transformation stress ratio [24], which is typically about 60% of the critical state stress ratio for Malaysia kaolin clay and Singapore upper marine clay. As shown in Fig 10, the mean effective stress for the Malaysia kaolin clay is approximately 80% lost after 100 cycles loading [23]. To identify the stress state of soil surrounding the spudcan footing during cyclic rocking, a small strain finite element simulation of a single cycle of large amplitude rocking on the spudcan was performed. This method has been demonstrated to provide a reasonable estimation on Yang’s [18] centrifuge model test results regarding the short-term and long-term spudcan fixities [22].

**Fig 11:**
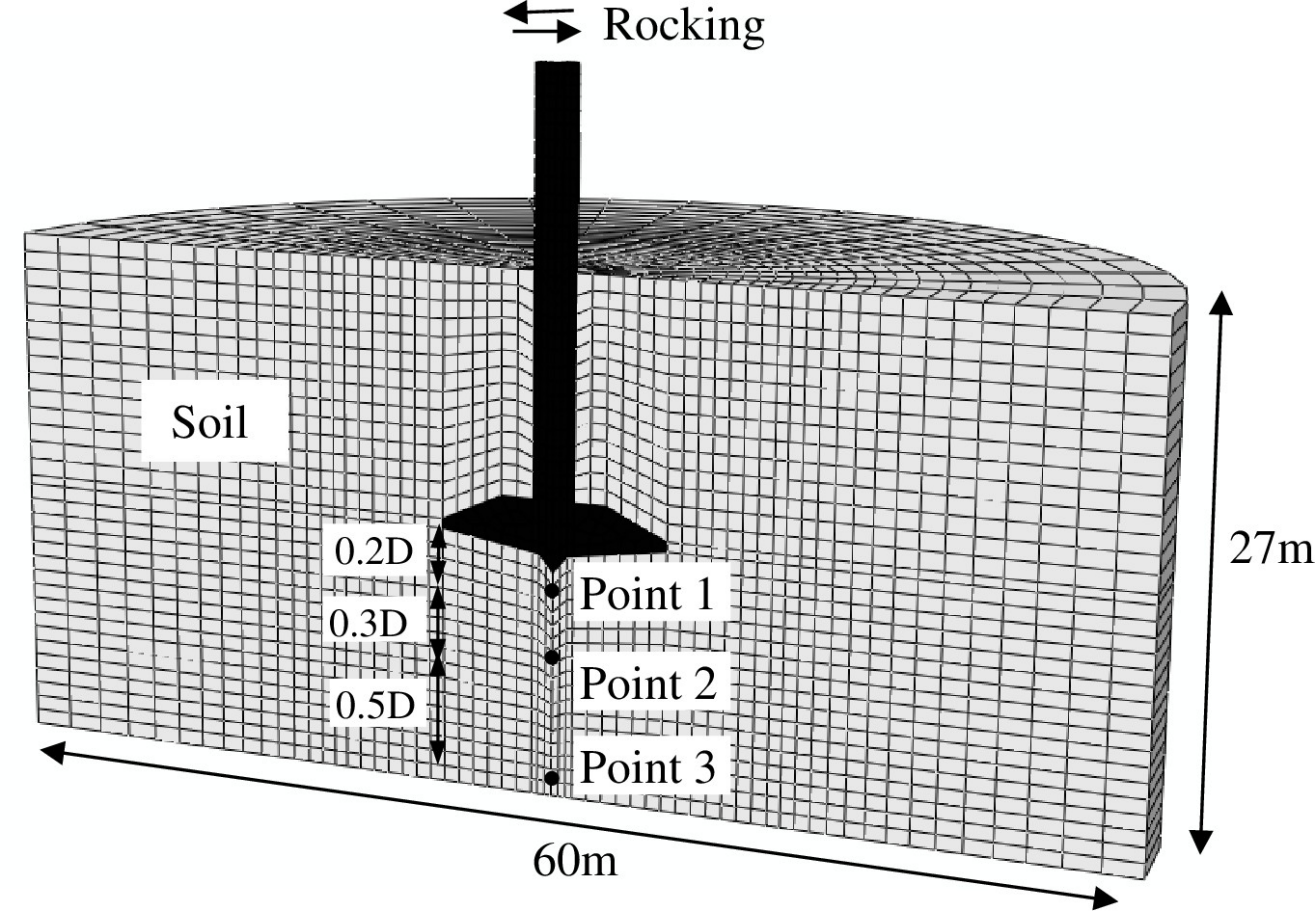
3D finite element mesh.

**Fig 12:**
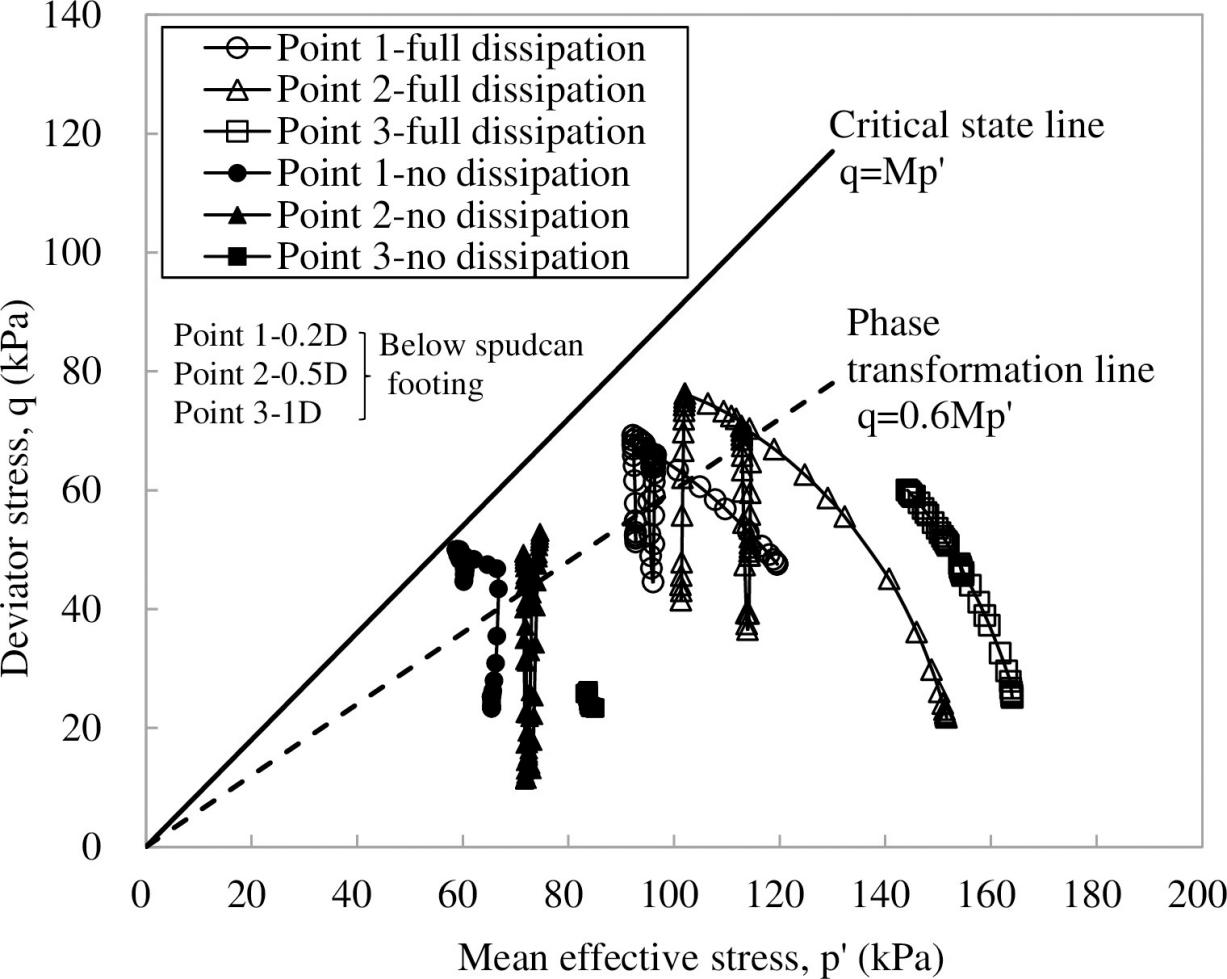
Computed stress paths of soil beneath spudcan footing during the 1^st^ cyclic rocking.

Fig 11 presents the three dimensional (3D) finite element mesh, its boundary lengths and boundary conditions coincide with those used in Yang et al.’s [17] centrifuge model test. In general, the modelling methodology in 3D is the same as that described in 2D finite element analysis. Four stages were involved in the 3D finite element analysis, i.e. penetration-unloading-consolidation-rocking. To replicate centrifuge model tests by Yang et al. [17], the spudcan was initially wished-in-place at a depth of 13.6 m, this was followed by an additional penetration of 1.5m. After that, the spudcan preload at 15.1m was half removed before commencing consolidation. For “full-dissipation” test, a consolidation time of about five years was prescribed; for “no-dissipation” test, rocking was conducted immediately after unloading with no consolidation being allowed. To simulate the large amplitude cyclic rocking, a maximum lateral distance of 1m was applied to the spudcan. Fig 12 shows that the computed stress ratios of soil elements within 0.5D beneath the spudcan footing can reach 94% and 83% of the critical state stress ratio, for “no-dissipation” test and “full-dissipation” test after the first rocking cycle, respectively. Both of these values are sufficient to generate large excess pore pressure with accompanying loss of soil strength and stiffness. Hence, a conservative approach may need to be taken into account for such cyclic loading effects.

## 6. Conclusions

The forgoing discussion explored the possible causes on additional spudcan settlements after the completion of preloading in the Gulf of Mexico sites, the findings show that full cavity collapse could only explain part of the measured additional settlements. Consolidation settlement is likely to account for a small proportion of the spudcan settlement, indeed probably less significant than that caused by cavity collapse. Centrifuge cyclic rocking tests demonstrate that large spudcan settlement can still occur if the spudcan is subjected to large amplitude cyclic loading, even after a 50% removal of the maximum preloading and the yield envelope was not exceeded. This leads to the possibility that stiffness and strength degradation of the soil in the vicinity of the spudcan footing and the leg might have occurred due to cyclic loading. The centrifuge results also show that the lattice leg has a significant effect on reducing the spudcan settlement during cyclic loading, possibly by taking up some of the moment arising from the loading on the jack-up rig [18]. In view of these findings, a conservative approach is recommended in instances where large amplitude cyclic rocking, such as that arising from storm loading, is expected shortly after preloading.

## Notations

A_a_: area ratio, ratio of the cross-sectional area of lattice leg to the maximum spudcan footprint
d: spudcan penetration depth, determined from the lowest point of the largest spudcan cross-sectional area to the mudline
D: spudcan diameter at the largest cross section
e: opening ratio, ratio of the area of the openings at the sides of the lattice leg to the total area of the lattice sides
H_max_: cavity depth determined from the mudline to the bottom of the cavity
H_ave_: averaged cavity depth with a cross sectional area of the maximum spudcan footprint
H_i_: cavity depth inside the lattice leg
H_o_: cavity depth outside the lattice leg
K_o_: coefficient of earth pressure at rest
k: coefficient of permeability
M_1_: measured maximum spudcan bending moment at the 1^st^ rocking cycle
M_2_: slope of critical state line
p': mean effective stress
q: deviator stress
s_u_: soil undrained shear strength
Tc: normalized consolidation time
t: consolidation time
V_ult_: maximum preload
V: vertical load hold during consolidation and cyclic rocking
V_s_: spudcan volume
w_cav_: spudcan settlement due to cavity collapse
w_con_: spudcan settlement due to consolidation
w_cyclic_: spudcan settlement due to cyclic loading
w: measured additional settlement
α: the ratio of the vertical load hold during consolidation over the maximum preload
λ: slope of isotropic normal compression line
κ: slope of isotropic swelling and recompression line
Γ: specific volume of soil at critical state (*p*' = 1*kPa*)
*υ*': effective Poisson’s ratio
*γ*': soil effective unit weight
*σ*_*v*_': soil effective vertical stress
*φ*': soil critical state friction angle
